# Cleansing Teeth

**Published:** 1897-08

**Authors:** Albert H. Brockway

**Affiliations:** Brooklyn, N. Y.


### CLEANSING TEETH.[1]

BY ALBERT H. BROCKWAY, M.D.S., BROOKLYN, N. Y.

I WAS asked not long since, by the publisher of a dental periodical, to give him for publication my method of cleansing teeth. As this seems to indicate an interest in the subject, I will set forth in brief my practice in this respect,—not assuming it to be different or better than that of others, but such as serves my purpose.

I will say, however, as an excuse for speaking on what may perhaps be considered a trivial matter, that I believe it is one to which many dentists give too little attention, and yet it seems to me that few of our operations are more generally beneficial or more highly appreciated by our patients.

In my own practice I make it preliminary to all other operations for several reasons: it familiarizes one with the character of the mouth and the condition of the teeth, and, also,—and this is important with timid or inexperienced patients,—it serves as an excellent and comparatively gentle introduction to the more severe operations which are to follow.

Having seated the patient in the chair, my assistant—a young lady, by preference, always—fastens around the neck a large napkin for the purpose of protecting the clothing from being soiled, often supplementing this with a light rubber apron if thought necessary,—a precaution much appreciated by fastidious persons.

Lightly smearing the lips and corners of the mouth with refined vaseline to prevent chafing, I then proceed with appropriate scalers to remove from the teeth every particle of tartar or other deposits, being greatly aided by my assistant, who holds back the lips and cheek, and illuminates the mouth with a small mirror or the electric lamp, frequently washing the loosened accretions with tepid water from a syringe. A few drops of listerine, or some of the analogous preparations, added to the water give a grateful, soothing freshness to the mouth, sweetening the breath, and rendering the operation more agreeable to all concerned.

My next step is to thoroughly polish all accessible surfaces of the teeth with a wheel-brush, of suitable stiffness, run by the dental engine, and charged with pulverized pumice-stone moistened with water or listerine. Should there still remain, as is often the case, persistent stains, I follow the brush with buffers or points of moose-

---

[1] Read before the New York Institute of Stomatology, April 6, 1897.

hide, or rubber charged like the brush, and sometimes rendered more efficient by the use of a little tincture of iodine or peroxide of hydrogen. In extreme cases, where the stain is *very* persistent, I moisten the polisher with a drop of dilute phosphoric acid with much advantage.

All the exposed portions of the teeth having been cleansed and polished, there still remain the proximal surfaces scarcely touched, and these are extremely difficult to reach, especially if the teeth are in close contact. Having made sure of the possibility of getting at these with any polishing agent, however fine, by first passing thin metallic strips between the teeth, I then proceed to use fine tape or floss silk charged with the pumice-powder, drawing it back and forth with a sawing motion, and bending it around to reach every part of the proximate surfaces.

I am free to say, however, that for this part of the operation I have never found anything so efficacious and satisfactory as what is known and sold at the dental depots as dental fibre, or tucum, a tropical production said to be taken from the palm-leaf and charged naturally with silex like the cortex of a reed, and which, consequently, can be used either wet or dry. This is so fine and strong that with care it can be readily drawn between the teeth, however close they may be, and, given the proper motion, will quickly and effectively remove all stains, leaving the surfaces beautifully polished and clean.

